# Meta-analysis and Systematic Review of Skin Graft Donor-site Dressings with Future Guidelines

**DOI:** 10.1097/GOX.0000000000001928

**Published:** 2018-09-24

**Authors:** Arman T. Serebrakian, Brent B. Pickrell, David E. Varon, Amin Mohamadi, Mark W. Grinstaff, Edward K. Rodriguez, Ara Nazarian, Eric G. Halvorson, Indranil Sinha

**Affiliations:** From the * Department of Surgery, Division of Plastic Surgery, Brigham & Women’s Hospital, Harvard Medical School, Boston, Mass.; † Center for Advanced Orthopaedic Studies and Carl J. Shapiro Department of Orthopaedic Surgery, Beth Israel Deaconess Medical Center, Harvard Medical School, Boston, Mass.; ‡ Departments of Biomedical Engineering, Chemistry, and Medicine, Boston University, Boston, Mass.; § Department of Orthopaedic Surgery, Yerevan State Medical University, Yerevan, Armenia; ¶ The Plastic Surgery Center, Asheville, N.C.

## Abstract

**Background:**

Many types of split-thickness skin graft (STSG) donor-site dressings are available with little consensus from the literature on the optimal dressing type. The purpose of this systematic review was to analyze the most recent outcomes regarding moist and nonmoist dressings for STSG donor sites.

**Methods:**

A comprehensive systematic review was conducted across PubMed/MEDLINE, EMBASE, and Cochrane Library databases to search for comparative studies evaluating different STSG donor-site dressings in adult subjects published between 2008 and 2017. The quality of randomized controlled trials was assessed using the Jadad scale. Data were collected on donor-site pain, rate of epithelialization, infection rate, cosmetic appearance, and cost. Meta-analysis was performed for reported pain scores.

**Results:**

A total of 41 articles were included comparing 44 dressings. Selected studies included analysis of donor-site pain (36 of 41 articles), rate of epithelialization (38 of 41), infection rate (25 of 41), cosmetic appearance (20 of 41), and cost (10 of 41). Meta-analysis revealed moist dressings result in lower pain (pooled effect size = 1.44). A majority of articles (73%) reported better reepithelialization rates with moist dressings.

**Conclusion:**

The literature on STSG donor-site dressings has not yet identified an ideal dressing. Although moist dressings provide superior outcomes with regard to pain control and wound healing, there continues to be a lack of standardization. The increasing commercial availability and marketing of novel dressings necessitates the development of standardized research protocols to design better comparison studies and assess true efficacy.

## INTRODUCTION

Split-thickness skin grafts (STSGs) are an integral part of the reconstructive armamentarium and serve a fundamental role in the treatment of burn, traumatic, and chronic wounds. Consisting of the epidermis with varying thicknesses of underlying dermis, a STSG offers several benefits beyond simple wound coverage.^1–7^ According to the Healthcare Cost and Utilization Project, over 160,000 skin grafts are performed annually in nearly 1 out of 3 burn hospitalizations.^8^ Their use is popular, as they are relatively easy and expedient, provided adequate donor tissue and a healthy recipient bed are available.

Once an STSG is harvested, an iatrogenic wound remains at the donor site that heals by reepithelialization from underlying dermal appendages over 7–10 days.^9^ Not infrequently, patients report high levels of pain at the donor site(s) resulting in lengthier hospitalizations, increased analgesic consumption, and impairment in functional activity that may delay overall recovery and early mobilization.^10–15^ Consequently, palliation of donor-site pain via different dressing preparations is a subject of clinical interest and ongoing debate.

In recent years, there has been an increasing number of commercially available dressings for STSG donor sites.^16–23^ Dressings are categorized as moist (eg, Aquacel, Kaltostat, Mepitel, Tegaderm) and nonmoist (eg, Xeroform, paraffin-impregnated gauze), depending on their ability to retain moisture upon application. Moist dressings provide an environment that prevents desiccation and are nonadherent to the wound bed, whereas nonmoist dry dressings possess no barrier to contain the extracellular fluid within the wound.^2,24^ In clinical practice, the choice to use moist versus nonmoist dressings largely remains based on surgeon preference without clear evidence-based consensus from the literature.

Three systematic reviews comparing different donor-site dressings have been published over the last 20 years.^2,25,26^ In the interim, new dressings have been introduced and higher quality comparison studies have been published that warrant revisiting this topic to include newer evidence.

## METHODS

### Literature Review

A systematic review was performed using PRISMA guidelines.^27^ Comprehensive literature searches were conducted in the PubMed/MEDLINE, EMBASE, and Cochrane Library online databases in June 2017 by 2 independent reviewers (A.T.S. and B.B.P.). The following key words were used independently and in combination to identify full-text articles published in English between 2008 and 2017: “skin graft,” “donor,” “dressing.” This time range was chosen to allow focus on data published since the previous review article. Articles were considered if they were comparative studies evaluating different STSG donor-site dressings in adult human subjects. Abstracts, case reports/series, studies with fewer than 10 subjects, review articles, surveys, letters to the editor, and meeting proceedings were excluded. Articles comparing STSG donor-site dressings in pediatric patients, and articles comparing secondary dressings, topical creams, sprays, or ancillary interventions (eg, ultrasound, extracorporeal shock wave therapy, active micro-current application, intradermal injections) were also excluded. Following database query and duplicate study removal, the titles and abstracts of the remaining preliminary studies were reviewed. Cross-referencing of included studies was then performed. Any disagreement was resolved through involvement of the senior author (I.S.).

Descriptive outcomes collected for each study included the following: year of publication, study type, number of subjects, follow-up duration, subject age, sex, diagnoses, donor dressings, location of donor site(s), and primary outcome measures (pain, reepithelialization, infection rate, cosmetic outcome, and cost).

### Quality Assessment

Methodological quality of randomized control trials (RCTs) was assessed using the Jadad Scale/Oxford Quality Scoring System. This system provides a method of identifying potential levels of bias by scoring each study with regard to randomization, blinding, intrastudy withdrawals and drop-outs.^28^ One independent reviewer (D.V.) scored each eligible study included in this review.

### Data Synthesis and Meta-analysis

A meta-analysis was performed for pain among articles that reported Visual Analog Scale (VAS) scores. Mean VAS, SD, and the number of patients in each group (moist and nonmoist) were used to calculate the Hedges’ g as an indicator of effect size for each study. We combined continuous assessments of pain using Hedges’ g, which is a “bias adjusted” measure of the Standardized Mean Difference and expresses the intervention effect in standard units rather than the original units of measurement.^29^ Meta-analysis was not performed for the remainder of outcome measures due to heterogeneity in study designs and lack of standardized outcome measurements.

If data were presented in 95% confidence intervals, the SD was calculated with the following formula: SD = 95% CI/1.96 × √n. When the median and range were reported for continuous outcomes, the mean and SD were estimated by assuming that the mean was equivalent to the median and that the SD is a quarter of the range. If no SD was given, the SD was estimated as half of the mean value. Hedges’ g also can be calculated with the proportion of “pain free” patients in each study arm or with the mean difference correspondent *P* values between 2 study groups.^30^

To address heterogeneity among individual studies, Cochran’s Q statistic and I^2^ were calculated. A *P* value of 0.10 or less was set to determine statistical significance.^31^ In the case of significant heterogeneity, a random effect analysis was employed. Otherwise, a fixed effect analysis was employed. To evaluate for the presence of publication bias, the Begg-Mazumdar test (with a *P* < 0.05 indicating statistically significant) was used.^32^

A time-point subanalysis was performed. Postoperative pain measurement time points were categorized into 3 groups: less than 4 days, 4–7 days, and 8+ days postoperative. Whenever multiple measurements in a time span were reported, we used the measurements from the longest follow-up time.

## RESULTS

### Literature Search and Study Characteristics

**Fig. 1.**
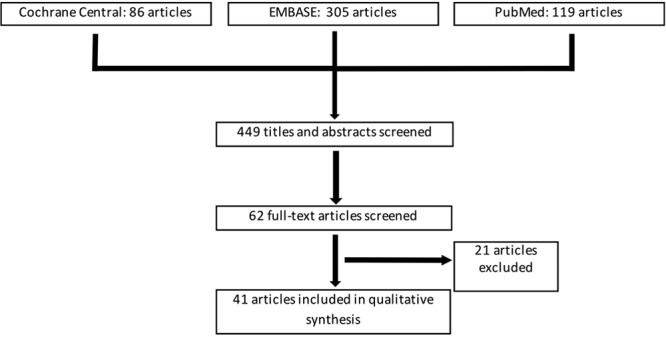
Flow chart for included studies.

**Fig. 2.**
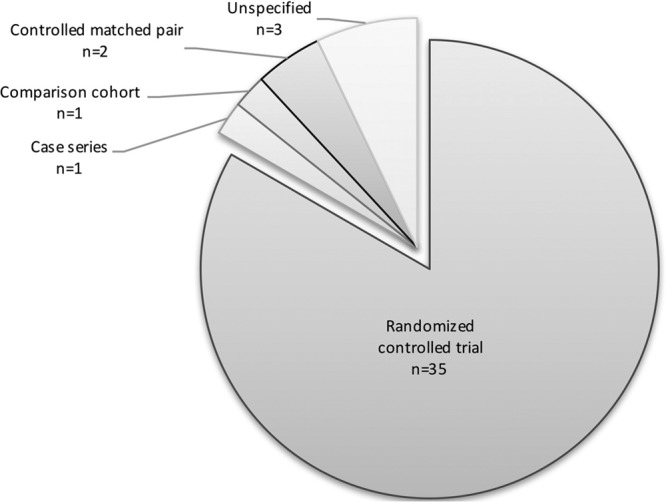
Study types that met inclusion criteria.

The literature search identified 510 articles (Fig. 1). After review and implementing exclusion criteria, 41 articles were included (Table 1). The majority of included articles were prospective RCTs (35 of 41 studies, Fig. 2). The remaining articles included prospective case series (1 article), comparison cohort clinical trial (1 article), prospective controlled matched pair studies (2 articles), or unspecified (3 articles). Patient diagnoses were described in 23 of 41 articles and included patients with burn injuries, chronic ulcers, traumatic wounds, wounds resulting from tumor resection (skin, bone, or unspecified), and extremity fasciotomies. A total of 44 different dressing types were evaluated and compared. Moist versus nonmoist (22 articles), moist versus other moist (22 articles), and nonmoist versus other nonmoist dressings (1 article) were compared. Four articles compared multiple different dressing categories (eg, both moist versus nonmoist and moist versus moist).

**Table 1.**
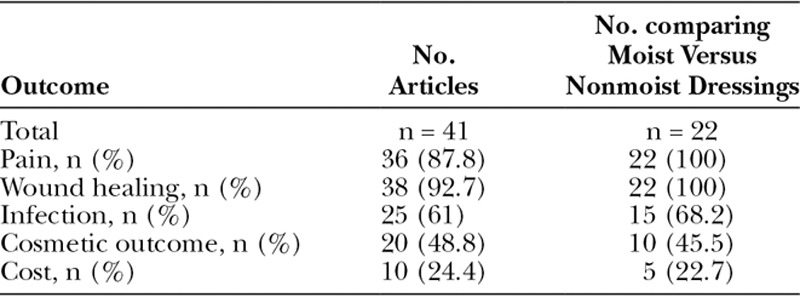
Number of Included Articles by Specific Outcome Measures

Patient demographics, including age and sex, were reported in 33 of 41 studies. Donor-site locations were also reported in 33 of 41 articles, with the lateral thigh donor site utilized most frequently. Other donor sites included back (2 articles), chest (1 article), arm (1 article), lateral trunk (1 article), buttock (1 article), and lower leg (1 article). Mean follow-up duration ranged from 9 days to 1 year and was either predetermined or set after study commencement (eg, time to complete reepithelialization).

**Fig. 3.**
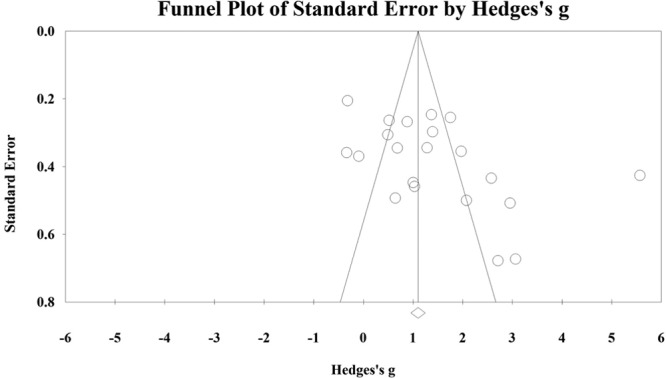
Funnel plot of standard error indicating absence of significant publication bias.

Mean Jadad score for all included studies was 1.54 ± 1.14. The funnel plot (Fig. 3) was not asymmetrical indicating the absence of publication bias. Similarly, Mazumdar test assessing publication bias was not significant (*P* = 0.085).

### Pain

**Fig. 4.**
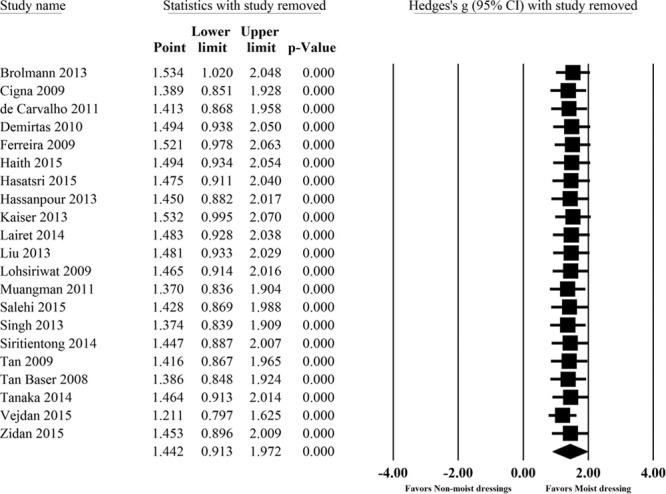
Meta-analysis of pain scores comparing moist vs. nonmoist dressings.

Pain was evaluated in 36 of 41 articles by various assessment methods (Table 2). Comparison of pain between moist and nonmoist dressings was performed in 22 articles, with 19 articles (86%) reporting lower pain scores with moist dressings. The remaining 3 articles reported no difference in pain between moist and nonmoist dressings. Sufficient data for quantitative analysis comparing pain intensity between moist and nonmoist dressings was reported in 21 articles. Eight of these articles did not report time of pain assessment and were therefore only entered in the overall analysis. The remaining 13 articles were included in a subgroup analysis based on time of pain assessment. The meta-analysis showed patients with moist dressings had significantly lower pain at all time points compared with nonmoist dressings (Fig. 4). Moist dressings appeared to be most effective in short-term assessments (Standardized Mean Difference = 1.32; 95% CI, 0.49–2.14); however, there was no significant difference in size of the effect among 3 assessed time points (*P* = 0.69; Table 3).

**Table 2.**
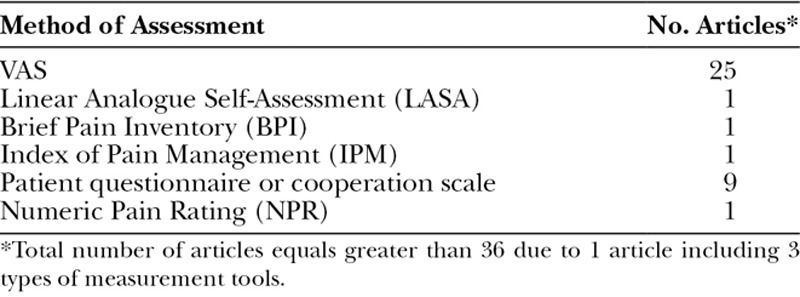
Pain Outcome Assessment Methods Used in the Included Articles

**Table 3.**
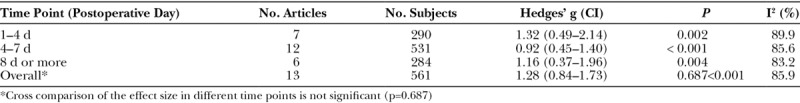
Subanalysis of Postoperative Pain Comparing Moist and Nonmoist Dressings at Different Time Points

### Wound Healing

Reepithelialization rate and/or quality of healing between dressing types was reported in 38 of 41 articles. Comparison between moist and nonmoist dressings was performed in 22 articles, with 16 articles (73%) reporting a better reepithelialization rate and/or quality with moist dressings. Five articles reported no difference in reepithelialization rate and/or quality, and 1 article reported better reepithelialization with a nonmoist dressing.

Donor site wound healing was defined in most articles as time elapsed between STSG harvest and complete donor-site reepithelialization (30 articles). In the remainder of articles, healing was defined by the amount of reepithelialization at donor sites at a given time point (7 articles) or average per day reepithelialization (1 article). Complete reepithelialization was determined by spontaneous detachment of the applied dressing or by clinical examination.

### Infection

Infection rate was reported in 25 of 41 articles and was defined by the presence of clinical signs or symptoms (eg, wound site erythema, purulence, and/or exudate). Overall, infection rates were low. Seven articles reported no infections, and the highest reported infection rate was 24%. The comparison of infection rates between moist and nonmoist dressings was reported in 15 articles, with 3 articles reporting higher infection rates with nonmoist dressings, 1 article reporting higher rates using a moist dressing, and the remainder (73%) reporting either no infections or no difference. Differences were not reported to be statistically significant.

### Cosmetic Outcome

Cosmetic outcome was reported in 20 of 41 articles with the majority of articles reporting no difference in cosmetic outcome between different dressing types. Comparisons between moist and nonmoist dressings were reported in 10 articles. Three articles (30%) reported better cosmetic outcomes using moist dressings, 2 articles (20%) reported better outcomes using nonmoist dressings, and 5 articles (50%) reported no difference. Assessment was performed using the modified Vancouver Scar Scale (VSS), Patient and Observer Scar Assessment Scale (POSAS), wound evaluation by clinicians and/or patients, or photo review by clinicians (Table 4). Two articles did not specify cosmetic assessment methods.

**Table 4.**
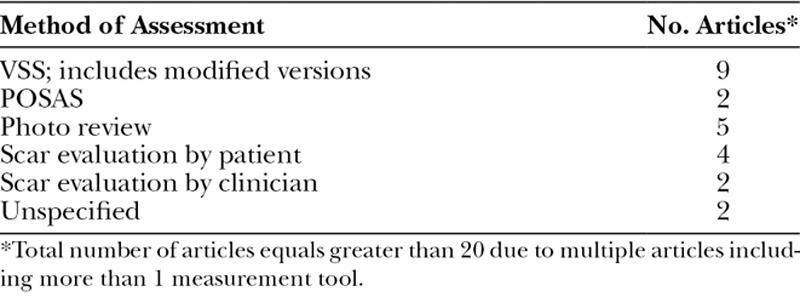
Cosmetic Outcome Assessment Methods Used in the Included Articles

### Cost

Descriptive comparisons of cost were performed in 10 of 41 articles with 5 articles (50%) comparing moist and nonmoist dressings. Two articles (40%) concluded moist dressings to be more cost effective. One article (20%) concluded nonmoist dressing to be more cost effective, and 2 articles (40%) found no difference. Cost comparison was performed using either cost per square centimeter of dressing, nursing labor and/or time spent by staff, or amount of waste produced.

## DISCUSSION

Despite continued publication of RCTs and the development of novel dressings, the optimal STSG donor-site dressing choice remains unclear. Our article builds upon the existing literature by providing an updated review and a new meta-analysis on pain scores using validated guidelines. We are able to associate moist dressings with better pain scores and wound healing, whereas infection rate, cosmetic outcome, and costs do not significantly differ between moist and nonmoist dressings. In addition, our review provides novel recommendations for future studies.

Traditionally, dressings for STSG donor sites are categorized as moist and nonmoist. Further categorization includes occlusive (eg, hydrogels, hydrocolloids), semiocclusive (eg, polyethylene film), or “exposure” dressings—where occlusive dressings completely seal off wounds and surrounding tissue from air, fluids, and possible contaminants.^33^ Both occlusive and semiocclusive dressings are included in the moist subgroup in this review.

Of the 22 articles comparing pain between moist and nonmoist dressings, pooled data show significantly lower pain scores with moist dressings. Additionally, pain was significantly lower with moist dressings at all postoperative time points in the subanalysis performed. Evaluation of reepithelialization involved various methods such as time elapsed until spontaneous detachment of the dressing or serial subjective evaluations by an experienced clinician. A number of studies evaluated reepithelialization rates (expressed as a percentage of total wound area) at a predetermined follow-up time point. The majority of articles reported moist dressings associated with improved wound healing.

Infection rates, cosmetic outcomes, and cost were reported in fewer articles. Infection rates were very low overall, and differences between dressing types were minor with no strong evidence to support the use of a specific dressing type. Cosmetic outcomes, reported in almost half of the included studies, found no clear evidence supporting the use of moist versus nonmoist dressings. Eleven of the 20 articles reporting cosmetic outcomes used validated scar outcome scales such as VSS or POSAS. Importantly, the VSS does not take patient perception into account, whereas POSAS incorporates subjective patient assessments.^34^ Finally, studies either reported cost in terms of direct monetary dressing value, or by incorporating variables such as patient length of stay and nursing labor costs for a more comprehensive cost assessment. When factoring all variables, there remains weak evidence to support the use of a specific dressing.

Three previous systematic reviews on STSG donor-site dressings have been published (Table 5).^2,25,26^ Rakel et al.^25^ reviewed 33 studies published between 1968 and 1996 and reported outcome measures including pain, infection, healing quality/rate, and cost. Major drawbacks of this review were that no definitive search criteria were described, and no quality assessment tools were utilized for the included studies. In 2003, Wiechula^26^ compared moist with nonmoist STSG donor-site dressings through evaluation of 58 studies and found that moist dressings were associated with faster healing, lower infection rates, and decreased pain scores. However, no direct time limits were reported in their literature search. To describe study quality, a checklist was developed by the authors based on the work of the Cochrane Collaboration and Centre for Reviews and Dissemination. These 2 reviews concluded moist dressings have superior outcomes with regard to pain, infection, and healing.

**Table 5.**
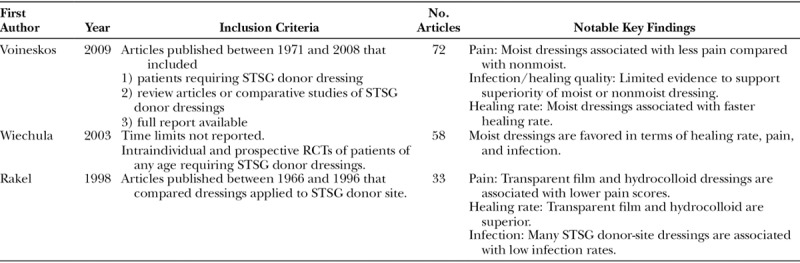
Previously Published STSG Donor-site Dressing Review Articles

Most recently, Voineskos et al.^2^ published a systematic review whose methodology adhered to established guidelines.^35^ They evaluated 72 studies published between 1971 and 2008 meeting specific inclusion and exclusion criteria. All methods for selecting studies were outlined, and the authors concluded moist dressings were associated with overall lower pain. In contrast to Wiechula,^26^ the authors did not find evidence to support moist dressings for reducing infection rates. Due to the heterogeneity of the studies included in their review, a meta-analysis was not performed, and study quality using the Jadad scale found low overall quality among the included studies (average score, 1.7 ± 0.7).

In all previous reviews, study quality was inherently poor due to methodological heterogeneity, poorly defined primary outcomes, and little use of existing validated measurement scales for pain and patient quality of life. Our review revealed similar methodological issues with large variations in outcome assessment tools. Specifically, pain outcomes were measured using various scales ranging from VAS to patient questionnaires. In addition, the articles in our review seldom used quality of life metrics or considered treatment intervention from the patient’s point of view.

The inherent weakness of our review is the heterogeneity and overall low quality of the included studies. The mean Jadad score for included studies was 1.54 ± 1.14 with scores ranging from 0 to 3. It was impossible for articles to achieve a maximum Jadad score of 5, because double-blinding these studies is not possible. Lack of consistency in outcome measures and poor study designs continue to plague the literature, although certain improvements are evident. Increased utilization of validated pain and scar scales are seen.^19,22,23,36–53^ Vast differences among the included dressings weaken this review. Well-established dressings are being compared with novel dressings being studied for the first time without standardized means for comparison.

### Novel Dressings

Systematically reviewing the literature and the inclusion of the most recently published work strengthen this review. Innovative dressing materials developed recently range from bioelectric dressings, bilayered silk gelatin, dressings with novel ointments including Triterpine, and new dermal substitutes. In a recent study surveying burn-care specialists, the “ideal” burn wound dressing was described as having properties of nonadhesion, absorbency, and antimicrobial activity.^54^ Goertz et al.^18^ describe a solidifying gel that dissolves according to temperature, providing a more user-friendly interface for patients with superficial wounds. Specifically, their novel gel is liquid at room temperature and hardens to a gel consistency at or above normal body temperatures resulting in lower pain and superior results in terms of staining, leakage, and odor when compared with silver sulfadiazine gauze. Another promising dressing recently described in nonhuman studies includes a gelling dendritic hydrogel-based dressing possessing thioester linkages that is able to dissolve on demand.^55–58^ The ability to apply a gel that solidifies within several minutes simplifies the dressing application process significantly. In vivo model studies have shown these gels achieve effective hemostasis and prevent infection while providing a moist environment for wound healing. Furthermore, a powerful feature is the ability for clinicians to dissolve the dressing on-demand for atraumatic dressing removals. Antibacterial chitosan-based gelling fiber dressings (Opticell Ag+) have also been introduced recently, providing a moist, conformable, highly absorbable dressing with antimicrobial activity to limit dressing changes and alleviate pain.^59^ Finally, stem cell therapy and lasers round out innovations that are serving as the frontier for burn and wound care.^60,61^

### Future Studies

Despite a plethora of studies, the literature on STSG donor-site dressings continues to be plagued by inconsistencies in research methodology. This literature review identified significant methodological issues including lack of standard study design and outcome criteria. Level I evidence is easily attainable. Future RCTs should use validated assessment instruments to report all outcome measures, ideally incorporating patient-reported outcome measures. Trials should be targeted toward patients with similar indications and common disease patterns. Postoperative dressing protocols and length of follow-up should be standardized. Finally, observational cohorts may be designed to identify predictors of treatment outcomes.

## CONCLUSIONS

Moist dressings provide superior outcomes with regard to pain and wound healing. However, there continues to be a lack of standardization for performing comparison studies between different dressing types. The increasing number of novel dressings necessitates the development of standardized research protocols to design better comparison studies. Given the clinical need for STSG donor-site dressings, significant opportunities exist for the continued development of new dressings, along with clinical trials using defined quantitative metrics of success, to improve patient care.
